# ASO Author Reflections*:* Rationale to Routinely Retest ER, PR and HER-2/neu Receptor Status in Residual Disease After Neoadjvuant Chemotherapy

**DOI:** 10.1245/s10434-024-16020-2

**Published:** 2024-08-06

**Authors:** Soumy Gottipati, Macy Goldbach, Julia Tchou

**Affiliations:** 1 Department of Surgery, University of California San Francisco, San Francisco, CA USA; 2 Division of Breast Surgery, Department of Surgery, Perelman School of Medicine, University of Pennsylvania, Philadelphia, PA USA

## Past

Pathologic complete response (pCR) after neoadjuvant chemotherapy has long been established as a biomarker associated with excellent prognosis. For those without pCR, prognosis correlates with residual cancer burden (RCB),^1^ which stratifies the extent of residual cancer in breast and axilla into ascending categories of RCB I, II, and III, which in turn correlates with worse prognosis.^2^ Although RCB is an excellent prognostic marker, it does not take into account the possible impact of receptor profile change in residual disease. With recent advances in targeted therapies, especially HER2-directed therapies,^3,4^ the frequency and clinical significance of receptor status change in residual disease is unclear.

## Present

We performed a retrospective study to evaluate the frequency and clinical significance of receptor profile changes in patients with residual disease after neoadjuvant chemotherapy (NAC) treated between 2009 and 2022 at two academic hospitals located on the East and West Coasts, respectively.^5^ Receptor profile retesting was not uniform. Retesting was performed in 352/392 (90%) patients at the West Coast site, while retesting was only performed in 90/241 (37.3%) patients at the East Coast site, with an overall retesting rate of 61.4% (259/422) [*p* < 0.0001]. The disparate retesting rate is most likely due to uneven implementation across multiple clinical sites within the East Coast site. We found that a clinically significant receptor profile change, defined as a change in breast cancer subtype post-NAC, was noted in 47/259 patients (18.1%) and was especially common (17/52, 32.7%) among those with pre-NAC HER2+ disease. Conversion from HR+/HER2+ to triple-negative breast cancer (TNBC) was significantly associated with worse disease-free survival (hazard ratio 36.7, confidence interval 2.2–611; *p* = 0.01). These results highlighted (1) receptor profile retesting variability and the need to uniformly implement receptor status retesting for all patients with residual disease after NAC; and (2) prognostic significance of receptor subtype change in residual disease.

## Future

Receptor retesting in residual disease after NAC should be standard practice. Inclusion of breast cancer subtype change in residual disease in RCB classification may strengthen its prognostic value and warrants further studies. A changed receptor subtype in residual disease post-NAC presents a unique opportunity for further research to enhance our understanding of disease heterogeneity’s impact on outcomes. For patients with pre-NAC non-TNBC converting to TNBC, it is unclear whether adjuvant chemotherapy with or without immune checkpoint blockade therapy may improve outcomes. For those with pre-NAC HER2+ disease converting to post-NAC HER2− disease, there is evidence to support the continuation of trastuzumab deruxtecan in the adjuvant setting.^6^ For those with pre-NAC HER2-0 disease converting to post-NAC HER2-low disease, the benefit of trastuzumab deruxtecan or other HER2 drug conjugate is unknown. Clinical trials to evaluate the effectiveness of these treatment strategies are needed.
